# RIG-I and TLR-7/8 agonists as combination adjuvant shapes unique antibody and cellular vaccine responses to seasonal influenza vaccine

**DOI:** 10.3389/fimmu.2022.974016

**Published:** 2022-11-08

**Authors:** Sonia Jangra, Gabriel Laghlali, Angela Choi, Raveen Rathnasinghe, Yong Chen, Soner Yildiz, Lynda Coughlan, Adolfo García-Sastre, Bruno G. De Geest, Michael Schotsaert

**Affiliations:** ^1^ Department of Microbiology, Icahn School of Medicine at Mount Sinai, New York, NY, United States; ^2^ Global Health and Emerging Pathogens Institute, Icahn School of Medicine at Mount Sinai, New York, NY, United States; ^3^ Graduate School of Biomedical Sciences, Icahn School of Medicine at Mount Sinai, New York, NY, United States; ^4^ Department of Pharmaceutics, Ghent University, Ghent, Belgium; ^5^ Department of Microbiology and Immunology, University of Maryland School of Medicine, Baltimore, MD, United States; ^6^ University of Maryland School of Medicine, Centre for Vaccine Development and Global Health (CVD), Baltimore, MD, United States; ^7^ Department of Medicine, Division of Infectious Diseases, Icahn School of Medicine at Mount Sinai, New York, NY, United States; ^8^ The Tisch Cancer Institute, Icahn School of Medicine at Mount Sinai, New York, NY, United States

**Keywords:** influenza vaccine, adjuvant, ADCC, T cell, antibody class switching, HA stalk, neuraminidase

## Abstract

Influenza vaccine effectiveness could be improved by combination with an adjuvant with the potential to enhance the host-vaccine response both quantitatively and qualitatively. The goal of this study was to explore a RIG-I agonist (SDI-nanogel) and a TLR7/8 agonist (Imidazoquinoline (IMDQ)‐PEG‐Chol) as adjuvants, when co-administered with a licensed quadrivalent inactivated influenza vaccine (QIV), and to determine the role of these adjuvants in directing helper T (Th) cell responses for their role in the immunoglobulin (Ig) class switching. Administration of QIV with the two adjuvants, individually or combined, resulted in enhanced HA-specific serum ELISA IgG titers, serum hemagglutination inhibition (HAI) titers and splenic T cell responses as examined by IFN-γ and IL-4 enzyme-linked immunosorbent spot (ELISPOT) assays, 4-weeks post-prime and post-boost vaccination in BALB/c mice. While QIV+SDI-nanogel largely induced antigen-specific IgG1 responses, QIV+IMDQ-PEG-Chol predominantly induced IgG2a antibody isotypes post-prime vaccination, suggesting efficient induction of Th2 (IL-4) and Th1 (IFN-γ) responses, respectively. Combination of the two adjuvants not only skewed the response completely towards IgG2a, but also resulted in induction of HAI titers that outperformed groups that received single adjuvant. Moreover, enhanced IgG2a titers correlate with antibody-mediated cellular cytotoxicity (ADCC) that targets both the highly conserved H1 hemagglutination (HA) stalk domain and N1 neuraminidase (NA). A booster vaccination with QIV+IMDQ-PEG-Chol resulted in a more balanced IgG1/IgG2a response in animals primed with QIV+IMDQ-PEG-Chol but increased only IgG2a titers in animals that received the combination adjuvant during prime vaccination, suggesting that class switching events in germinal centers during the prime vaccination contribute to the outcome of booster vaccination. Importantly, IMDQ-PEG-Chol, alone or in combination, always outperformed the oil-in-water control adjuvant Addavax. Vaccine-induced antibody and T cell responses correlated with protection against lethal influenza virus infection. This study details the benefit of adjuvants that target multiple innate immune receptors to shape the host vaccine response.

## Introduction

Despite several vaccine candidates available on the market, influenza virus is responsible for severe illness in humans, with a substantial global death toll every year (https://gis.cdc.gov/grasp/fluview/flu_by_age_virus.html). Seasonal Influenza A virus (IAV) and influenza B virus (IBV) co-circulate in the human population and keep evolving with time. Vaccination against circulating IAV and IBV strains is the only effective way of protection against severe disease. However, due to antigenic drift in influenza viruses, vaccines need to be updated every year to protect against the circulating strains of the virus. Moreover, antibody responses induced by influenza virus vaccines are usually short-lived and less cross-reactive against antigenically drifted virus variants than those induced by a natural influenza virus infection (1). Additionally, vaccine-induced neutralizing antibody titers diminish over time, thereby affecting the extent of protection against infection during an entire influenza season and the subsequent seasons. Therefore, there is an urgent need for a better cost-effective influenza vaccine which can induce antigenically broader and long-lasting immune response.

Protective anti-influenza immunity often correlates with antibody responses to influenza surface glycoproteins, particularly hemagglutinin (HA), the main antigenic determinant on the surface of both influenza virus and infected cells. Different immunoglobulin G (IgG) subclasses produced by class-switched B cells help in opsonization and virus neutralization and hence, facilitate viral clearance from the host. In addition, these antibodies can also mediate several host effector functions. For instance, IgG2a engages with complement system components through their Fc region (2). IgG2a also engages in high affinity interactions with Fc receptors on immune cells which can result in antibody-dependent cell-mediated cytotoxicity (ADCC) as well as antibody-dependent cellular phagocytosis (ADCP) by phagocytosing cells like macrophages (3–8). IgG1 and IgG2a interact with Fc receptors on cells surface but with different affinities, and are different between humans and mice (5, 9, 10). Murine IgG2a, which is functionally similar to human IgG1, has been previously reported to enhance viral clearance in mice as an “effector antibody” (11, 12). Thus, a humoral response that provides broader protection to antigenic variants of the infecting virus, in addition to inducing a balanced antibody titers post-vaccination is desired in order to combine the protective effect of neutralization and opsonization.

Currently, several influenza vaccines are being used for humans which include inactivated virus split virus, and subunit vaccines consisting of IAV and IBV components (13, 14). Inactivated influenza virus vaccines are reported to induce a humoral response with high IgG serum levels in both mice and humans but poorly induce cell-mediated immunity (15, 16). Natural influenza infection induces Th1-type immune responses, characterized by more IgG2a antibodies in mice, which enhance viral clearance (11, 17–22). In contrast, the most commonly used split inactivated influenza vaccines (IIV), including trivalent inactivated vaccines (TIV) or quadrivalent inactivated vaccines (QIV), induce high IgG1 levels corresponding to a Th2-biased immune response (15, 16, 22, 23). Since most vaccines themselves induce IgG1 production, combining them with specific adjuvants can skew IgG2a/IgG1 ratios (24). In this perspective, many studies have been focusing on vaccine formulations with adjuvant mixtures to enhance a balanced IgG subclass response.

In this study, we formulated QIV from 2018-19 season with different adjuvants, individually and in combination, to examine the IgG subclass as well as T cell responses in an influenza vaccination/challenge mouse model. Specifically, we used an *in vitro* transcribed Sendai virus defective-interfering RNA [SDI-RNA; a RIG-I agonist (25)], and amphiphilic imidazoquinoline [IMDQ-PEG-Chol; TLR7/8 agonist (26)], as adjuvants for QIV. The antibody responses were further correlated with their cytokine profiles, T cell induction and protection against A/Singapore/GP1908/2015 (IVR-180), a matching strain to one of the QIV components.

## Results

### SDI-RNA formulation into nanogel for targeted and efficient delivery

**Figure 1:**
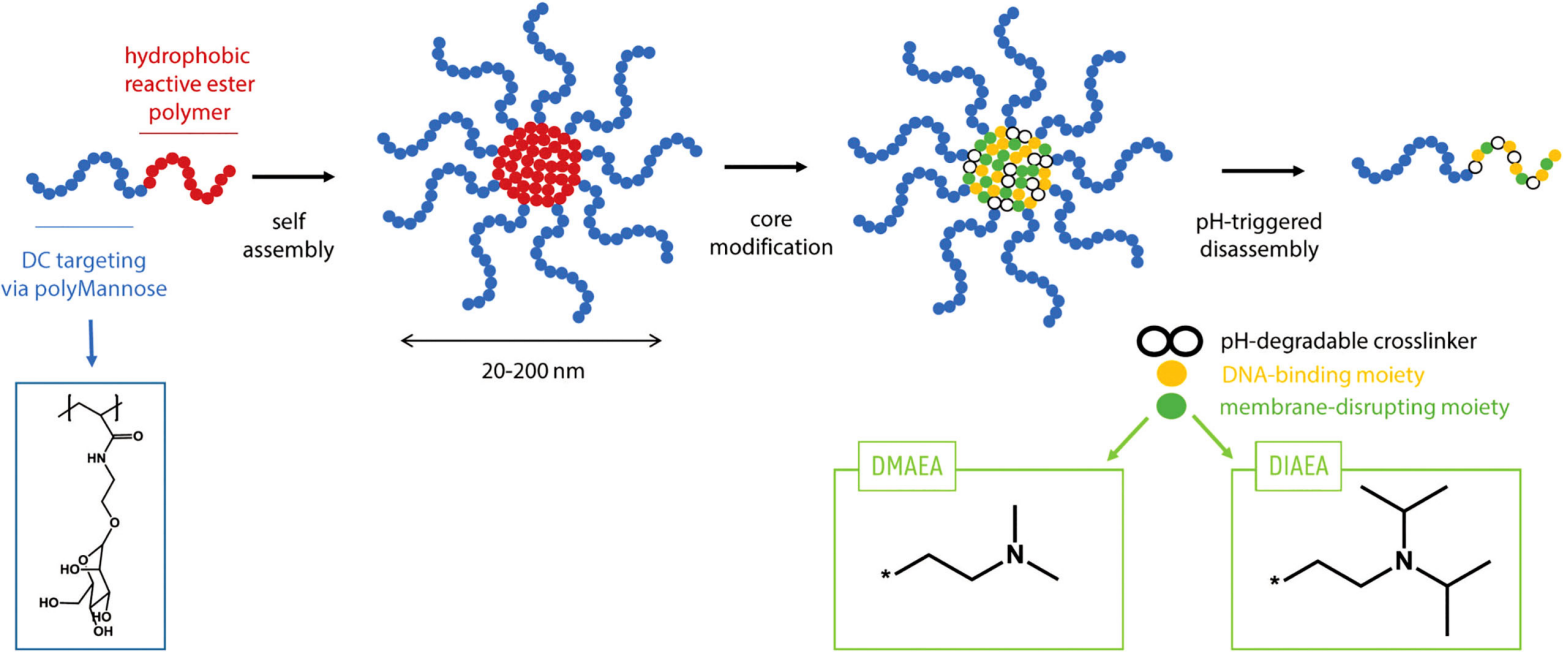
Principle of using nanogel for adjuvant formulation: Polymers consisting of a polyMannose chain which targets Dendritic cells and a hydrophobic reactive ester polymer which forms the positively charged core. The polymers can self-assemble in a solvent and disassemble upon endocytosis due to lower pH.

SDI-RNA (or SDI) has been characterized as a potent RIG-I agonist which induces type-I interferon responses (27). In this study, we used an *in vitro* transcribed SDI. Since RNA is very prone to degradation in physiological environment, for an efficient delivery of SDI *in vivo*, it has been combined with a nanogel reported previously by De Coen et al. (**Figure 1**) (28) These nanogels are based on core-crosslinked block copolymers composed of α,D-mannosylethyl acrylamide and pentafluorophenyl acrylate. The poly(α,D-mannosylethyl acrylamide) chain specifically targets dendritic cells. The poly(pentafluorophenyl acrylate) chains on the core are used for crosslinking with an acid-sensitive ketal containing bisamine and for introduction of ionizable N,N-Dimethylaminoethyl and N,N-Di-isopropylaminoethyl motifs which provide a cationic charge to the nanogel core, promoting electrostatic interaction with negatively charged SDI. Upon uptake by host cells, the decrease in pH triggers disassembly of the nanogel due to a pH-sensitive ketal crosslinker in the nanogel core, thereby releasing the SDI.

### SDI+nanogel and IMDQ-PEG-Chol are potent adjuvants for QIV

**Figure 2:**
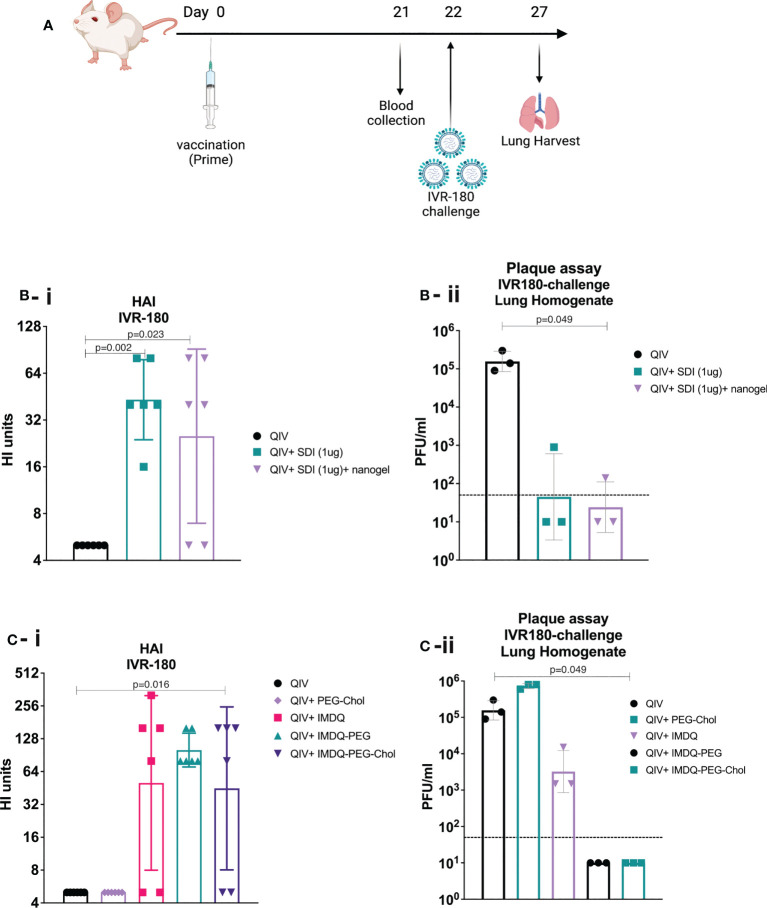
SDI+nanogel and IMDQ-PEG-Chol are potent adjuvants for QIV: **(A)** Schematic representation of the experiment (created with BioRender.com). **(A, B)** 6-8 weeks BALB/c animals (n=6/group) were vaccinated with unadjuvanted QIV (1.5μg HA equivalent per mouse) or combined with either 1μg SDI as adjuvant (with or without 4μg nanogel) or with 100μg of different forms of IMDQ. 3 weeks post vaccination, mice sera was examined for HAI titers **(B-i**, **C-i)** using 4HA units/well of IVR-180 virus. Consecutively, 3 animals from each group were challenged with 100LD_50_ of IVR-180 virus, and the lung virus titers were quantified by plaque assays on pre-seeded MDCK cells, five days post infection **(B-ii**, **C-ii)**. Each column represents the geometric mean ± geometric SD for respective groups. Statistical analysis was performed using two-sided unpaired T test. P values are calculated in reference to unadjuvanted QIV group for respective assays.

IMDQ-PEG-Chol is an amphiphilic formulation of the imidazoquinoline TLR7/8 agonist IMDQ with cholesteryl-poly(ethyleneglycol) conjugate which was recently reported by us (26). Contrary to unformulated soluble IMDQ, IMDQ-PEG-Chol shows limited systemic exposure and translocates efficiently to lymphoid tissue, thereby providing localized innate immune activation. To examine the humoral responses upon vaccine administration, 6–8-week-old female BALB/c mice were vaccinated intramuscularly once with unadjuvanted QIV or QIV combined with single or combined adjuvants. The experimental design is outlined in **Figure 2A**. The antibody responses were analyzed by hemagglutinin inhibition assay (HAI). The groups receiving QIV with SDI or SDI+nanogel showed high and almost comparable HAI titers, compared with the unadjuvanted QIV group (**Figure 2B-i**). The HAI titers were further correlated with protection against a lethal challenge of IVR-180 (100LD_50_), an IAV strain matching the H1N1 component of QIV vaccine. Single vaccination of naive mice with unadjuvanted QIV did not result in detectable HAI titers, suggesting that the mice did not seroconvert yet with single dose vaccination, so two doses might be needed for reliable HAI induction. As shown in **Figure 2B-ii**, mice vaccinated with QIV formulated with SDI, with or without nanogel, were found to have low viral titres in their lungs 5 days post-infection implying protection against influenza virus infection. Additionally, our previously described IMDQ-PEG-Chol (TLR7/8 agonist) (26), was also found to elicit high HAI titers in 4 out of 6 animals, similar to unmodified IMDQ (**Figure 2C-i**), but with better lung virus control (**Figure 2C-ii**) five days post-infection when compared with unadjuvanted QIV or QIV with unmodified IMDQ.

### SDI-RNA+nanogel and IMDQ-PEG-Chol induce type-I/III IFN responses *in vivo*

**Figure 3:**
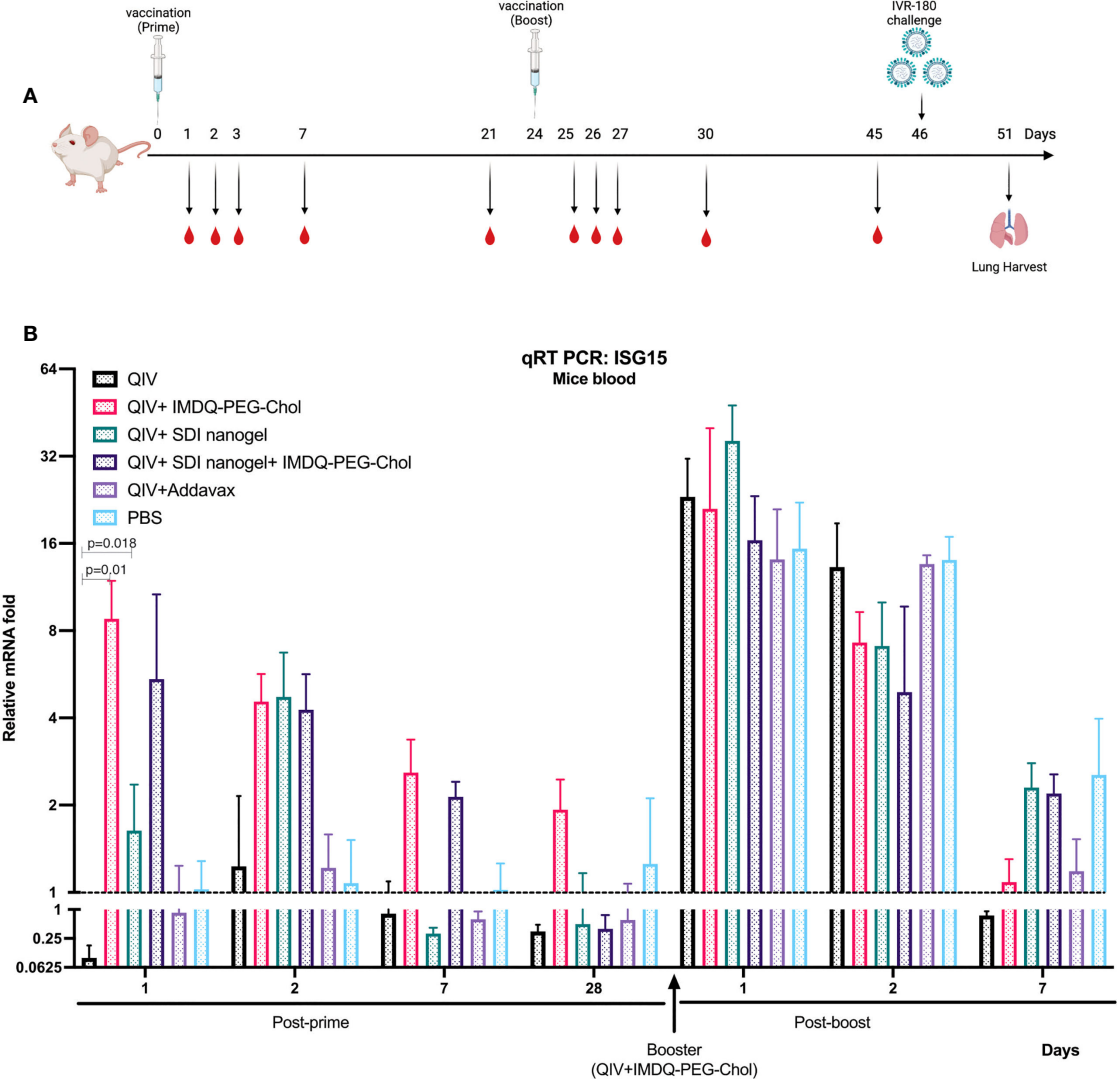
QIV combined with SDI+nanogel or/and IMDQ-PEG-Chol induces ISG15 expression: **(A)** Schematic representation of the experiment. 6-8 weeks BALB/c animals (n=4/group) were vaccinated with unadjuvanted QIV or combined with either SDI+nanogel and/or IMDQ-PEG-Chol and blood was collected at different time points for RNA analysis (created with BioRender.com). **(B)** Fold induction in ISG15 gene expression in mice from different groups, post-prime and post-boost vaccination. Importantly, all the animals received QIV+IMDQ-PEG-Chol as a booster, irrespective of the vaccination in prime dose. The fold changes are calculated with respect to the PBS group at each time point and represented as geometric mean for each group. GAPDH was used as internal control for the analysis. The error bars represent geometric SD for respective groups. Statistical analysis was performed using two-sided unpaired T test. P values are calculated in reference to unadjuvanted QIV group post-prime vaccination.

Since SDI is a RIG-I agonist, it can effectively activate downstream signalling pathway, thereby enhancing the production of Type-I/III interferon (IFN) and proinflammatory cytokines. IMDQ-PEG-Chol has already been shown to induce type-I IFN production in IFN-reporter mice in our previous study (26). To explore the short-term and long-term induction in innate immune responses by SDI+nanogel and IMDQ-PEG-Chol as adjuvants *in vivo*, BALB/c mice were vaccinated with QIV ± adjuvants, individually or in combination. Further, all animals receiving different vaccine-adjuvant combinations during prime dose, were boosted with the same QIV+IMDQ-PEG-Chol. Blood was collected at different time points post-prime and post-boost vaccinations (**Figure 3A**). Induction in type-I/III IFN was correlated with transcription of downstream interferon-stimulated gene, ISG15. As shown in **Figure 3B**, compared to the PBS group, the unadjuvanted QIV group did not induce ISG15 gene expression following a single prime vaccination. While SDI+nanogel induces ISG15 expression after 24 hr and peaks at 48 hr, IMDQ-PEG-Chol-mediated ISG15 gene expression, and by extension primary IFN induction, was enhanced within the first 24 hr of prime dose and became transient over the following days. ISG15 expression in the case of QIV+IMDQ-PEG-Chol seemed independent of its combination with SDI+nanogel implying that IMDQ-PEG-Chol itself is very potent in inducing primary IFN responses. Furthermore, all groups showed enhanced ISG15 gene expression immediately following the booster dose with QIV+IMDQ-PEG-Chol. More in-depth characterization of the cytokine/chemokine environment induced by IMDQ-PEG-Chol with and without SDI+nanogel is ongoing to better understand its potential or any possible long-term side effects as an adjuvant.

### Vaccination with SDI+nanogel and IMDQ-PEG-Chol result in strong IgG subclass switching

**Figure 4:**
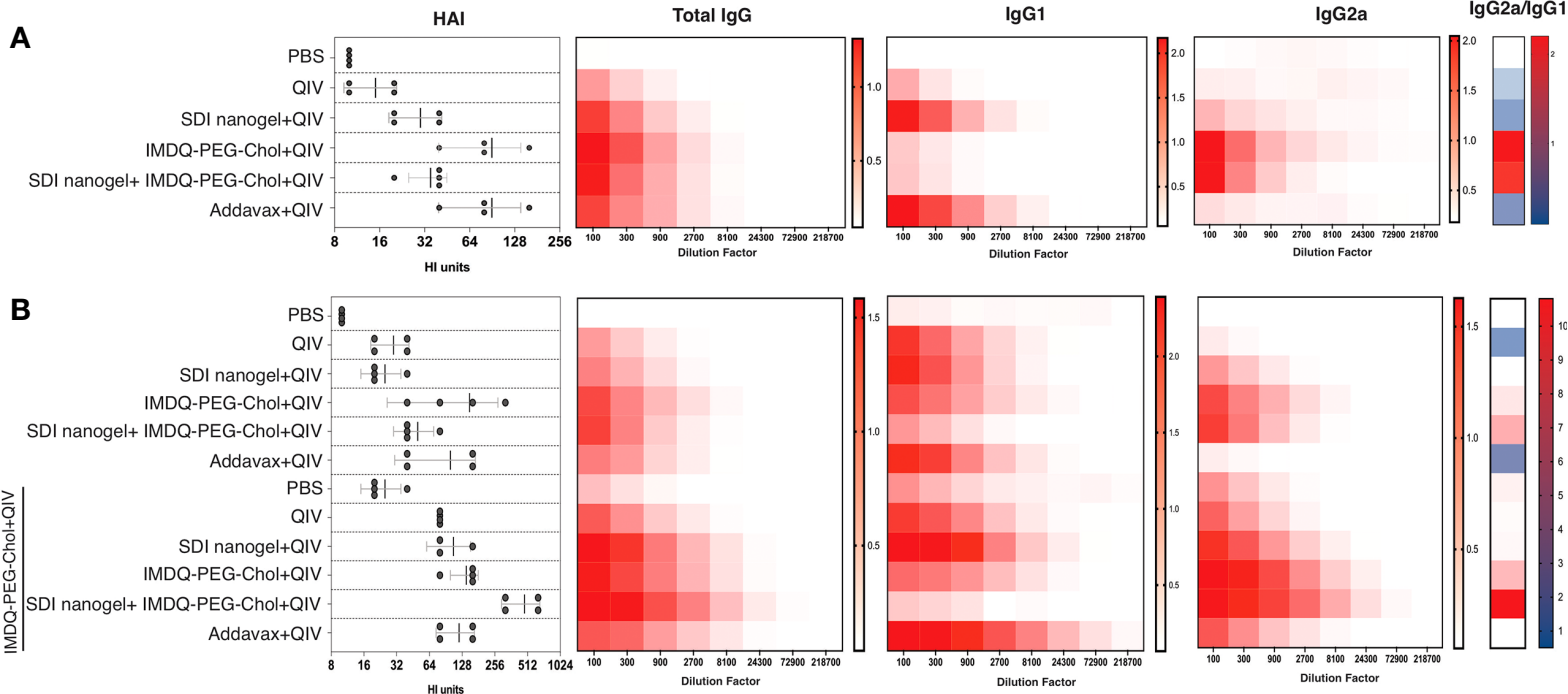
SDI+nanogel and IMDQ-PEG-Chol, individual or combined, define IgG subtype profile: Average OD_450_ ELISA values (n=4/group) plotted against serum dilutions, 4 weeks post-prime **(A)**, 8 weeks post-prime **(B)** which is 4 weeks post-boost for half of the mice as indicated **(B)**. HAI was performed using 4 HA units of IVR-180 virus (left top and bottom panels). Total IgG, IgG1 and IgG2a titers were quantified by ELISA with 3-fold serum dilutions starting with 1:100 and shown as heatmaps. The ratio of IgG2a/IgG1 was calculated based on the mean of area under the curve for individual animals in all groups. The HAI titers are represented as geometric mean ± geometric SD.

BALB/c mice have been previously reported to show enhanced Th2-type immune response with high serum IgG1 levels in response to inactivated or subunit influenza vaccines (22, 29). In this context, it was highly intriguing to explore the immunological profiles in response to QIV combined with SDI+nanogel and IMDQ-PEG-Chol as adjuvants, individually and in combination. 6–8-week BALB/c mice were vaccinated with unadjuvanted QIV and QIV in different combinations with SDI+nanogel and IMDQ-PEG-Chol. As shown in **Figure 4A** (and **Supplementary Figures 1A-i-iii**), following 4 weeks post-vaccination (or post-prime), QIV alone was found to induce predominantly IgG1 but serum titers remained very low. Addavax, a squalene-based MF59-like oil-in-water emulsion, was used as a reference for the study which was found to induce largely IgG1 antibodies with undetectable IgG2a levels in the serum. Similar to Addavax, SDI+nanogel combined with QIV largely enhanced IgG1 production while IMDQ-PEG-Chol was found to primarily induce IgG2a production in mice with very low levels of IgG1 4-weeks post-vaccination (**Figure 4B**). However, by 8 weeks post-prime, even in the absence of a booster injection, we observed influenza-specific IgG1 titers in the IMDQ-PEG-Chol group, resulting in a balanced IgG1/IgG2a response. When SDI+nanogel and IMDQ-PEG-Chol were administered together with QIV to test if the combination affects the IgG2a/IgG1 balance, we observed a response completely skewed towards IgG2a antibody subtypes. This is somewhat surprising, as we saw solely IgG1 levels promoted by SDI+nanogel alone. For this group, contrary to what we observed for IMDQ-PEG-Chol alone, the antibody profile remained IgG2a-skewed after 8 weeks of vaccination with a single dose (**Figure 4B** and **Supplementary Figures 1B-i-iii**). Experimental groups were split in two and half of the animals received a booster dose of QIV+IMDQ-PEG-Chol, intramuscularly 4-weeks post-prime vaccination. The purpose of administering the same booster (QIV+IMDQ-PEG-Chol) to all groups was to examine if the IgG1/IgG2a skewing is observed only in certain groups or all irrespective of the adjuvant combination in prime dose. As a result of the booster, all groups started to show IgG2a production, resulting in a more balanced IgG2a/IgG1 profile, except for the group which initially received combination adjuvants (IMDQ-PEG-CHOL+SDI/nanogel) with QIV during prime vaccination (**Figure 4B**). In this group IgG2a/IgG1 ratio remained completely skewed towards IgG2a.

As a surrogate for virus neutralizing antibody levels, hemagglutinin inhibition (HAI) assays were done with mouse sera collected at four (prime) and eight (prime and boost) weeks post-vaccination. Mice that received unadjuvanted QIV showed low HAI titers similar to the PBS/unvaccinated group. HAI titers with QIV+Addavax and QIV+IMDQ-PEG-Chol had the highest titers post-primer vaccination and QIV with SDI+nanogel resulted in intermediate HAI titers. HAI titers went up in all experimental groups that received a booster dose with QIV+IMDQ-PEG-Chol. The group that initially received QIV with combination adjuvant (IMDQ-PEG-Chol plus SDI+nanogel) and boosted with QIV+IMDQ-PEG-Chol developed the highest HAI titers among all groups, with the majority being IgG2a antibodies. This also correlated with the highest overall ELISA total IgG titers when calculated as area under curve (**Supplementary Figure 1**).

### QIV adjuvanted with IMDQ-PEG-Chol, alone or in combination with SDI+nanogel, induces antibody-dependent cell-mediated cytotoxicity (ADCC) in vaccinated animals

Vaccine-induced antibodies can provide protection during respiratory infection through mechanisms beyond virus neutralization (30–32). Effector cells, such as natural killer (NK) cells and monocytes/macrophages, express Fcγ receptors (FcγI-III in humans and FcγI-IV in mice) that bind to the Fc region of the antibodies with different affinities, initiating a signalling cascade which can activate release of cytotoxic factors that result in killing of the infected target cell. Murine IgG2a antibodies are considered effector antibodies, and can bind specific Fcγ receptors thereby, promoting ADCC efficiently (33, 34). To examine the levels of ADCC in vaccinated animals in different groups, we used a luciferase-reporter based ADCC assay as described in the methodology section. Importantly, we used target MDCK cells which express chimeric H6/1 HA on the surface (the stalk is derived from H1 HA but the head is from an exotic irrelevant H6 HA to which no cross-reactive antibody responses are expected) (35, 36), allowing us to evaluate the contribution of H1 stalk-specific antibodies present in vaccinated mouse sera to ADCC. This assay suggested that sera which resulted in positive ADCC activity, mainly targeted the highly conserved H1 stalk domain in HA, and therefore could contribute to antigenic breadth.

**Figure 5:**
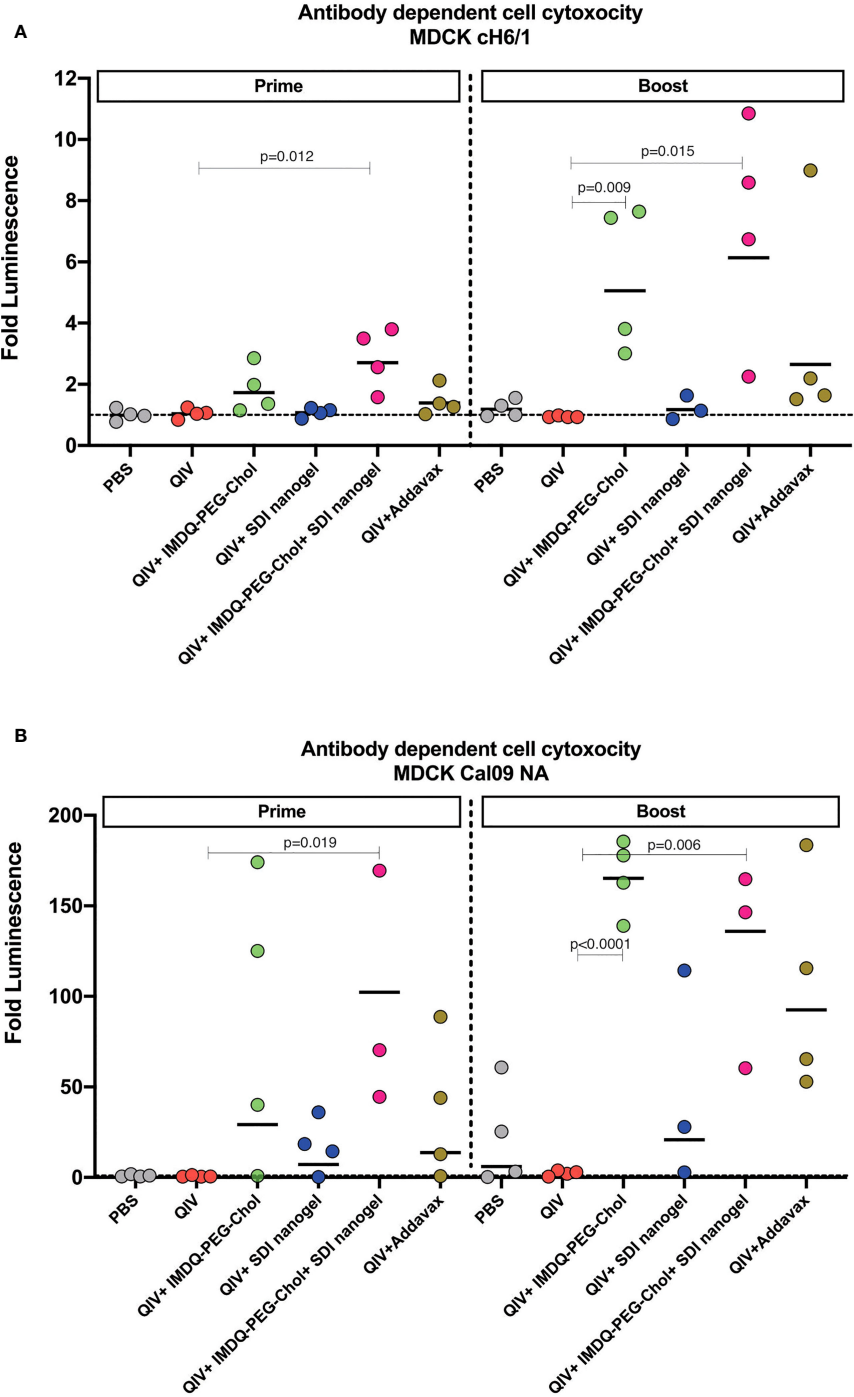
IMDQ-PEG-Chol enhances ADCC in vaccinated animals. Pre-seeded MDCK cells, stably expressing chimeric H6/1-HA **(A)** or Cal09-NA **(B)**, were incubated with post-prime or post-boost vaccination sera, followed by an incubation with effector Jurkat cells expressing FcγIV receptors. The readout was in terms of luminescence which was used to calculate the area under curve (AUC) for each sample against antibody dilutions. The ADCC activity is represented as fold luminescence based on fold change in AUC for individual animal in all groups in reference to the PBS group. The line in each group represents geometric mean of fold Luciferase activity and each dot corresponds to individual animal in the respective group. Luminescence levels reflect FcγR engagement and therefore are a surrogate for ADCC activity. Statistical analysis was performed using two-sided unpaired T test. P values are calculated in reference to respective unadjuvanted QIV groups.

As shown in **Figure 5A**, following 4-weeks post-prime vaccination, QIV alone, which was found to induce predominantly IgG1, resulted in low levels of H1/6 stalk-specific ADCC-inducing antibodies, almost comparable to the PBS group. Similarly, ADCC was very low in QIV+ SDI+nanogel group, correlating to high IgG1 but low IgG2a titers. On the other hand, groups that received QIV+IMDQ-PEG-Chol individually or in combination with SDI nanogel, showed higher ADCC correlating to higher IgG2a. ADCC activity in sera from animals which received QIV+IMDQ-PEG-Chol as a booster (4-week post-boost), followed similar trend as post-prime vaccination with IMDQ-PEG-Chol and IMDQ-PEG-Chol + SDI/nanogel outperforming the other experimental groups. Mice that received unadjuvanted QIV followed by a booster with QIV + IMDQ-PEG-Chol did not mount ADCC titers equally efficient as PBS mice boosted with IMDQ-PEG-Chol. We also detected Cal09-NA-specific ADCC-inducing antibodies (**Figure 5B**) and the groups receiving IMDQ-PEG-Chol as adjuvant, with or without SDI+nanogel, were found to have highest ADCC activity. Therefore, we conclude that vaccination with adjuvanted QIV allowed us to induce antibody responses that target conserved influenza antigens, more specifically the H1 HA stalk and the N1 neuraminidase.

### SDI+nanogel and IMDQ-PEG-Chol efficiently induce T cells responses

The cellular immune response can play an important role in protection by providing class switch help to B cells thereby shaping humoral responses, and through their roles in direct killing of infected cells (37, 38). CD4^+^ and CD8^+^ T cells along with innate immune cells such as dendritic cells (DCs), natural killer (NK) cells, and macrophages are components of cell-mediated immunity. Even though T cells themselves cannot recognize free pathogens, they can identify viral antigen presented in the context of molecules of the major histocompatibility complex on infected cells and exert effector cell functions such as the release of specific cytokines or a direct cytotoxic effect. CD4^+^ T cell or helper T (Th) cell subsets release cytokines such as IFN-γ and IL-4 corresponding to type-1 (Th1) and type-2 (Th2) helper T cells, respectively. In this regard, we investigated the release of IFN-γ and IL-4 from immune cells obtained from the major secondary lymphoid organ, the spleen, from BALB/c mice post-prime and post-boost vaccination, in presence and absence of specific antigen (either whole IVR-180 virus or H1 HA peptide) using ELISPOT assays.

**Figure 6:**
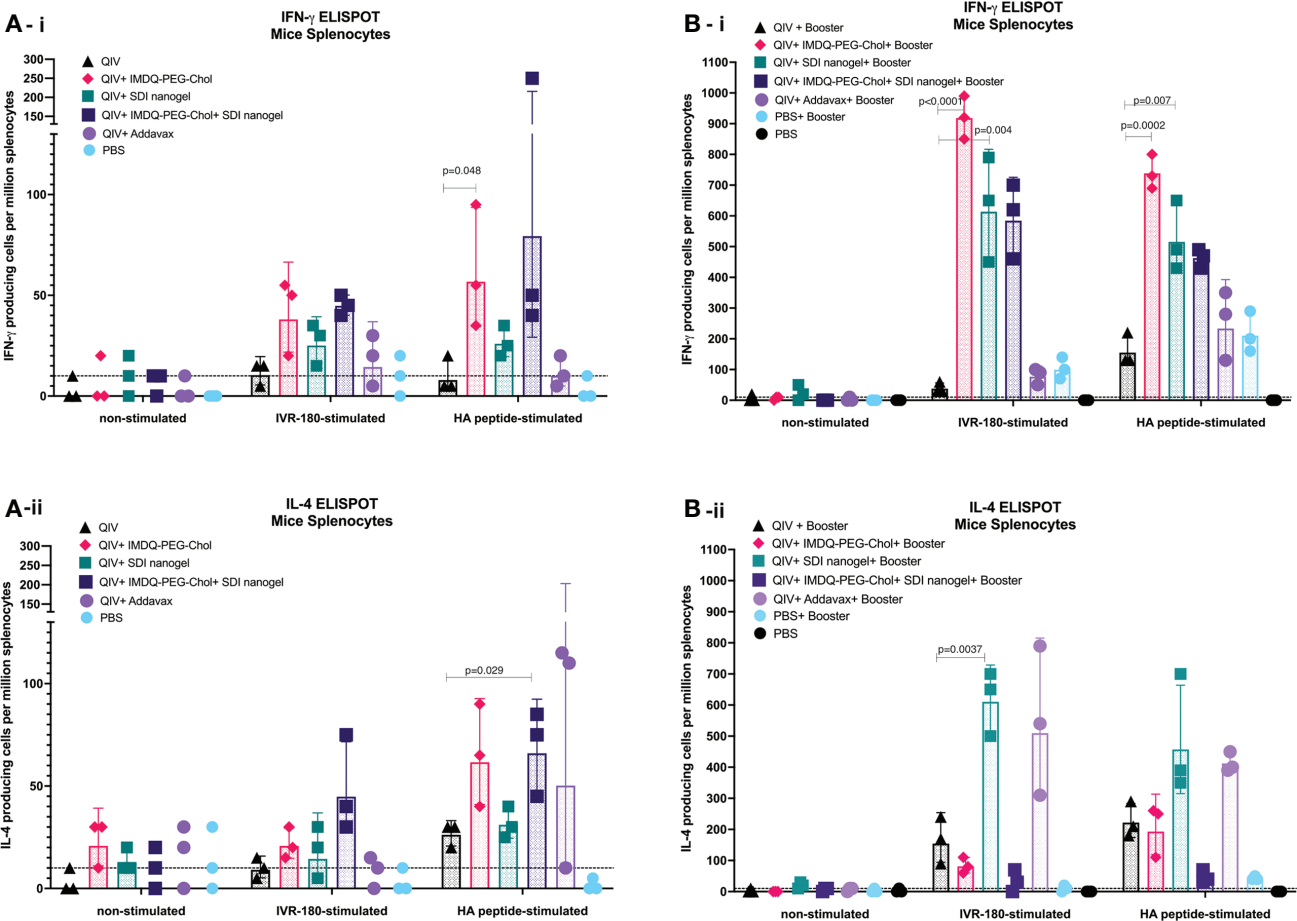
SDI+nanogel and IMDQ-PEG-Chol can effectively induce T cell responses in vaccinated animals: 6-8 weeks BALB/c mice were vaccinated with QIV with and without SDI+nanogel and/or IMDQ-PEG-Chol and spleens were harvested 10 days post-prime **(A)** and post-boost **(B)** to examine the T cell activation by IFN-γ **(A-i, B-i)** and IL-4 **(A-ii, B-ii)** ELISPOTs upon restimulation with H1-HA short-overlapping peptides. The results are represented as IFN-γ or IL-4 producing cells per million splenocytes (geometric mean ± geometric SD) for n=3 animals per group. The cut-off was set to 10 which indicates one spot in any well. Statistical analysis was performed using two-sided unpaired T test. P values are calculated in reference to respective unadjuvanted QIV group.

As shown in **Figures 6A-i/ii**, **B-i/ii**, corresponding to prime and booster vaccine dose respectively, antigen-specific IFN-γ or IL-4 release from the splenocytes was very low in the absence of a stimulant. Upon stimulation with specific H1-HA peptide or IVR-180 virus, the number of splenocytes producing antigen-specific IFN-γ were found to be higher in the mice that received QIV+IMDQ-PEG-Chol, with and without SDI+nanogel (**Figure 6A-i**), suggesting that the adjuvant efficiently drives a Th1 biased immune response. The overall number of cells producing IFN-γ post-prime vaccination was higher in all adjuvanted vaccine groups, with groups that received IMDQ-PEG-Chol and/or SDI/nanogel outperforming the reference group that received QIV+Addavax. Following a booster vaccination with QIV+IMDQ-PEG-Chol, the groups that received either or both of SDI+nanogel and IMDQ-PEG-Chol showed highest numbers of IFN-γ producing splenocytes (**Figure 6B-i**). Although the booster with QIV+IMDQ-PEG-Chol increased the number of IFN-γ producing splenocytes in mice that received QIV+Addavax as a primer vaccination, this number remained lower when compared with groups that were given SDI+nanogel, IMDQ-PEG-Chol or both during prime vaccination. Similarly, adjuvanted QIV groups were found to have a few IL-4 producing splenocytes post-prime vaccination (**Figure 6A-ii**). Once boosted with QIV+IMDQ-PEG-Chol, QIV with SDI+nanogel and Addavax receiving groups showed very high numbers of IL-4 producing cells implying a strong Th2 type immune response, which is in line with the dominantly IgG1 antibody titers measured in these groups (**Figure 6B-ii**). Mice that received QIV with a combination of IMDQ-PEG-Chol and SDI+ nanogel showed high IL4+ T cell numbers after prime, however this was reverted after boosting with IMDQ-PEG-Chol (**Figures 6A-ii, B-ii**).

### SDI+nanogel and IMDQ-PEG-Chol adjuvants potentiate vaccine responses upon QIV vaccination that correlate with protection against a lethal viral challenge with homologous influenza virus

**Figure 7:**
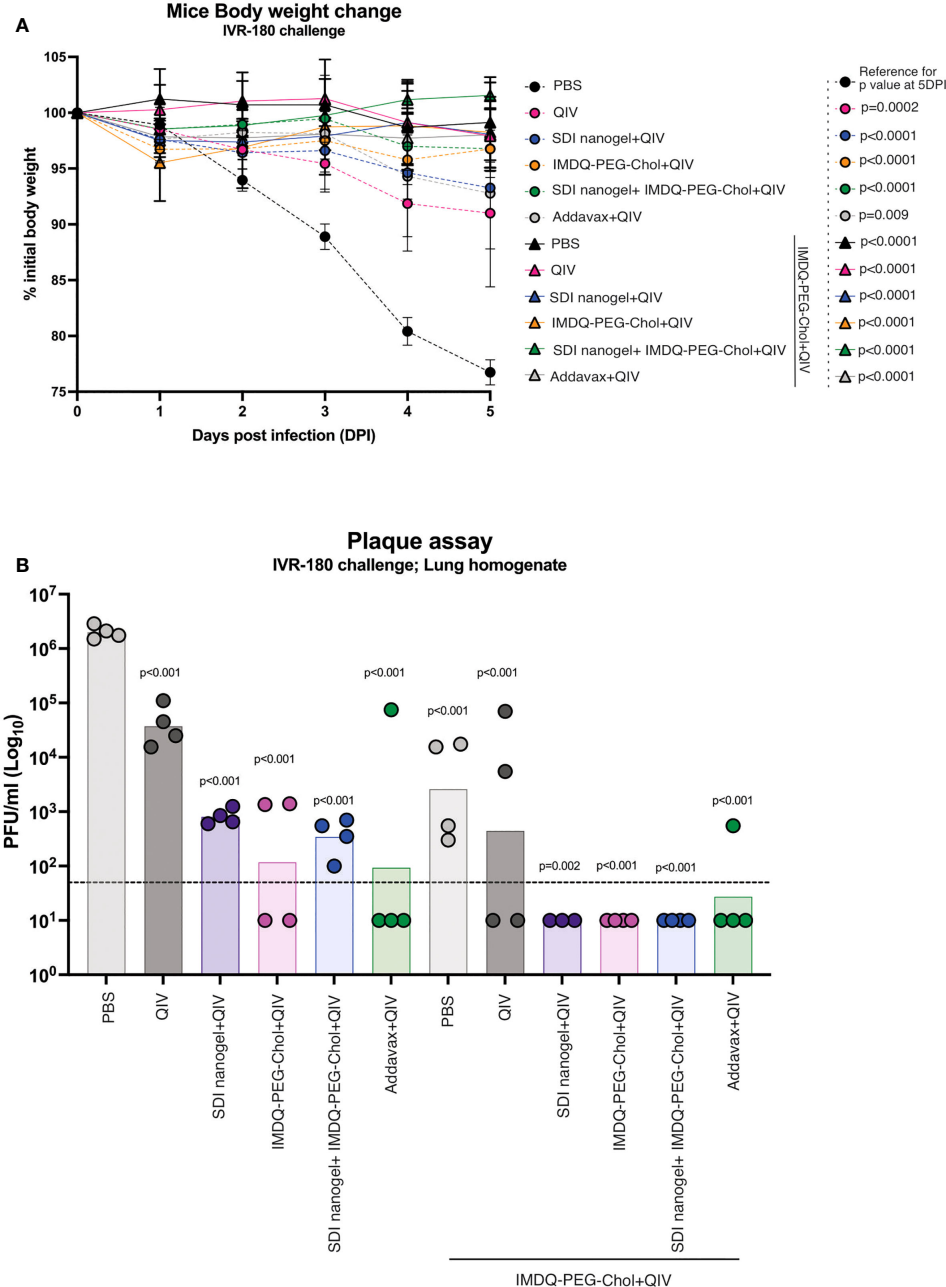
QIV combined with SDI+nanogel and/or IMDQ-PEG-Chol protects the animals against a lethal dose of IVR-180 virus: All unvaccinated and QIV ± adjuvant vaccinated animals were intranasally challenged with 100LD_50_ (18000 PFU/animal) of IVR-180 virus. **(A)** The body weights were recorded every day for 5 days post-infection and represented as percentage initial body weight. Day 0 represents the day of infection. **(B)** The viral lung titers were quantified by plaque assays on MDCK cells and are represented as Plaque‐forming‐unit (PFU)/ml for n=4 animals per group (geometric mean with geometric SD). Statistical analysis was performed using two-sided unpaired T test. The p values shown are calculated in reference to the PBS group which received no vaccination in both (**A**; 5 days post infection) and **(B)**.

To correlate the observed humoral and cell-mediated vaccine responses with protection, mice were challenged with a lethal dose of IVR-180 virus (100LD_50_). As shown in **Figure 7A**, vaccination resulted in protection from severe morbidity, whereas unvaccinated mice (PBS group) showed almost 25% body weight loss by day 5 of infection. All groups that received adjuvanted QIV once or twice showed <5% weight loss post-infection. Adjuvanted QIV also resulted in better control of lung virus titers (**Figure 7B**), and therefore correlates with enhanced vaccine responses observed in mice that received adjuvanted vaccine. Mice that received only a single vaccination dose with adjuvanted vaccine were more protected with lower viral titers than mice vaccinated with unadjuvanted QIV, but at the same time, they were less protected than mice that received a booster vaccination dose with undetectable lung virus titers.

## Discussion

Influenza vaccines have been available for multiple decades and can prevent severe disease. Yet, the currently licensed influenza vaccines are often only sub-optimally effective, and people still get sick and hospitalized upon influenza virus infections. This can be due to several reasons, like poor induction of neutralizing antibodies, antigenic mismatch between vaccine and circulating virus strains and waning immunity. There have been some advancements over the last century including the revision of trivalent inactivated influenza vaccines (TIV), which consists of two IAV and one IBV strain component, to quadrivalent inactivated influenza vaccines (QIV) formulation with addition of another IBV to tackle the evolving influenza B strains. Increasing the number of booster doses is another way to boost the immunity against the virus but is strategically and economically challenging. Alternatively, combining those vaccines with adjuvants to enhance host immune responses, seems a rather feasible and more effective option (29). Our study is based on combining QIV with different adjuvant formulations including SDI/nanogel and IMDQ-PEG-Chol to induce protective vaccine responses.

Unadjuvanted QIV results in modest serum IgG titers. These antibody titers remain low unless combined with adjuvants, as also shown in our and other previous studies in context of TIV or QIV vaccines (26, 39). Currently, a wide range of adjuvants are being studied for their immunogenicity in context of many vaccines (29, 40). Adjuvants are typically immune potentiators that are known to activate innate immune signaling cascades through activation of innate immune sensors. In our study, we show a role for two recently developed adjuvants, a TLR7/8 agonistic IMDQ-PEG-Chol and a RIG-I agonistic SDI-RNA in boosting and modulating both B and T cell mediated responses, correlating them with the level of protection against a hundred times half-lethal dose (100LD_50_) of a strain-matched IAV challenge virus (IVR-180).

While natural virus infection induces largely IgG2a responses in mice, which is indicative of a dominant Th1 response, the split inactivated vaccine has been previously reported to predominantly induce IgG1 antibodies in BALB/c mice suggestive of Th2 responses, which is also confirmed in our results (17, 18, 22, 23). We additionally show that combining QIV in different adjuvants will not only enhance the overall IgG titers but also induce specific subclasses of IgG depending on the type of adjuvant used and therefore the kind of PRRs (pattern recognition receptors) engaged. This may also be useful to enhance certain type of immune responses in immunocompromised individuals.

Although mice and human IgG subtypes (IgG1, IgG2a/c, IgG2b, IgG3 in mice and IgG1, IgG2, IgG3 and IgG4 in humans) differ in their structure and affinity towards Fc receptors (FcγRs) on the surface of immune effector cells, they are functionally homologous in terms of mediating effector functions such as ADCC (41). IgG1 in both mice and humans is mainly associated with a Th2 while IgG2a is associated with Th1 profile. Besides, both IgG1 and IgG2a are crucial to provide protective immunity against viruses (11, 19). This was confirmed in our study where mice vaccinated with adjuvanted vaccine showed lower lung virus titers implying a better control over virus infection after five days of infection, particularly after receiving a booster dose of QIV+IMDQ-PEG-Chol with lowest lung virus titers.

Depending on the adjuvant, specific antigen-presenting cells (APCs), B cells and Th cells can be activated, hence boosting either or both Th1 and Th2 responses (24, 42). Although those T cells do not target pathogens directly, they can exert effector functions together with releasing functional cytokines upon identifying the antigen-loaded/infected cells (43). Th1 cells secrete interleukin-2 (IL-2) and IFN-γ to enhance cell-mediated immune response while Th2 cells secrete IL-4, 5, 6 and 10 which regulates humoral responses by B cells (44). SDI, when mixed with nanogel, targets mainly DCs, which upon activation results in IgG1 responses corresponding to high Th2-associated cytokine levels. IMDQ-PEG-Chol, on the other hand, enhances Th1 type responses as suggested by IFN-γ cytokine release upon activation and correlates very well with IgG2a antibody response. Overall, these two adjuvants can target and activate different B cell and T cell subtypes and therefore, prove to be potent inducers and modulators of both innate and cell mediated immunity.

Although, in combination, these two adjuvants were expected to induce both IgG1 and IgG2a and therefore a balanced Th1/Th2 response, whereas IMDQ-PEG-Chol mediates IgG2a titers predominantly. The combination of these two adjuvants leads to minimal IgG1 responses and skewing the humoral vaccine response completely towards IgG2a. This skewing was further prominently observed when these groups were boosted with QIV+IMDQ-PEG-Chol. The animals receiving the combination adjuvants in prime dose showed robust IgG2a titers with minimal IgG1 response. This is somewhat contradictory to the dogma since this group actually mounted both a fairly strong IFN-γ and IL-4 T cell response after prime however this trend was reversed after booster vaccination with a post-boost T cell response predominantly characterized by IFN-γ secretion upon restimulation. The combination adjuvant group also showed the highest hemagglutination inhibition titers, high IFN-γ release from splenocytes upon re-stimulation, effective host effector functions (ADCC) all correlating with high IgG2a levels and high levels of protection against viral challenge.

Adjuvant admixing with QIV did not only result in higher antibody titers, but we also showed that adjuvanting QIV with IMDQ-PEG-Chol, SDI+nanogel or both resulted in higher ADCC activity targeting conserved epitopes within the influenza haemagglutinin as well as the N1 neuraminidase. Therefore, our novel adjuvant strategy results in enhanced antigenic breadth, which is important to provide longer protection against antigenically drifted influenza viruses.

Our results reveal a synergistic effect of simultaneous stimulation of RIG-I and TLR7/8, resulting in early class switching to uniquely IgG2a B cells. Therefore, we anticipate strong T cell involvement in germinal center reactions after vaccination. This will be the subject of follow up studies. We are aware that TLR7/8 signalling in mice cannot be directly translated to humans due to differences in their ligand-recognition function and expression pattern on cells in both species (45, 46). From our results, it can be anticipated that once the immune cells are primed and directed to a certain subtype response by a specific adjuvant system, it might be difficult to re-direct them to a different subclass response simply by changing the vaccine-adjuvant formulation. Therefore, we need in-depth studies in order to identify the deciding factor for germinal centers and its correlation with protection against infection for future vaccine formulations.

## Methods

### Reagents

A description of the reagents used in this study is outlined in **Table 1**.

**Table 1:** Source of reagents and kits used in the study.

Reagent	Brand	Catalogue number
DMEM	Corning	10013-CV
RPMI 1640	Gibco	22400089
Penicillin/streptomycin	Corning	30002-CI
Hygromycin	Thermofisher	10687010
RNase A	Ambion	AM2270
ITS liquid media supplement	Sigma	I3146
Goat HRP-conjugated secondary Anti-mouse IgG antibody	Abcam	Ab6823
Anti-mouse IgG1-HRP secondary antibody	Invitrogen	PA174421
Anti-mouse IgG2a-HRP secondary antibody	Invitrogen	A10685
Anti-mouse IgG(H+L)-HRP secondary antibody	Thermofisher	62-6520
TMB substrate	BD OptEIA	555214
KPL true blue substrate	Sera care	5510-0050
70μm strainer	BD	352340
Addavax	*In vivo*gen	Vac-adx-10
Peptivator H1-HA peptide	MiltenyiBiotech	130-099-803
Oxoid agar	Thermofisher	LP0028B
EMEM	BioWhittaker	12684F
TPCK	Sigma	T4376
10% methanol-free formaldehyde	Polysciences	040181
Ampliscribe T7-Flash transcription kit	Lucigen	ASF3257
Bio-Glo Luciferase Assay system	Promega	G7940
Mouse IFN-γ ELISPOT kit	RnD systems	EL485
Mouse IL-4 ELISPOT kit	RnD systems	EL404
cDNA reverse transcription kit	Thermofisher	4368814
Lightcycler SYBER Green I Master	Roche	14571520
ELISA NUNC-maxisorp plates	Invitrogen	44240421
Mouse Ribopure blood RNA isolation Kit	Invitrogen	AM1951

### Cell lines

Madin-Darby canine kidney (MDCK) cell line was cultured in Dulbecco’s Modified Eagle Medium (DMEM) supplemented with 10% Foetal bovine serum (FBS, Hyclone) and 1X penicillin/streptomycin. MDCK cells stably expressing cH1/6 (gift from the Krammer laboratory, Icahn School of Medicine at Mount Sinai) for ADCC were maintained in the same medium with Hygromycin.

### QIV vaccine

Quadrivalent Inactivated influenza vaccine (FLUCALVEX 2018/2019 season Lot 252681) was from Seqirus. The vaccine consisted of MDCK-grown Influenza A and B viruses- A/Singapore/GP1908/2015 IVR-180 (H1N1) (an A/Michigan/45/2015-like virus), A/North Carolina/04/2016 (H3N2) (an A/Singapore/INFIMH-16-0019/2016 -like virus), B/Iowa/06/2017 (a B/Colorado/06/2017-like virus) and B/Singapore/INFTT-16-0610/2016 (a B/Phuket/3073/2013-like virus).

### SDI-RNA

The SDI RNA was transcribed *in vitro* using AmpliScribe T7 high yield transcription kit from Lucigen according to manufacturer’s protocols. Quality control of SDI was assessed by IFN-β luciferase assay as shown in **Supplementary Figure 2**. RNase A treated or untreated SDI RNA was co-transfected with IFN-β-Luc reporter encoding the luciferase gene under the control of the IFN beta promoter and firefly Renilla luciferase, pSV-Rluc plasmid, for internal control, into HEK293T cells. 48 hours (hr) post-transfection, the cells were lysed, and luciferase activity was measured by adding Bio-Glo luciferase reagent using a BioTek plate reader. Live Sendai virus (SeV) infection was used as a positive control for the assay.

### Vaccine-adjuvant preparation and administration

For each animal, 1.5μg HA equivalent of QIV was mixed with 1μg SDI-RNA (with 40ug nanogel; 1:40 ratio) or 100 μg IMDQ-PEG-Chol (equivalent to 10 μg core IMDQ) or both and vortexed for 30 seconds (sec). Adjuvant doses were chosen based on our previously published work with these adjuvants (26, 27). Unadjuvanted or adjuvanted QIV was administered intramuscularly in a total of 100μl per mouse, divided equally over both hind legs. The control group were administered with equal volume of PBS instead of vaccine or vaccine/adjuvant mixture, divided over both hind legs. All animals received QIV+IMDQ-PEG-Chol as booster, irrespective of the prime vaccination.

### IVR-180 virus

150 PFU of the A/Singapore/GP1908/2015 IVR-180 (H1N1) was injected into the allantoic fluid of 8-days old embryonated chicken eggs and was incubated for 48 hr at 37°C followed by an overnight incubation at 4°C. The allantoic fluid, containing the virus, was carefully collected, briefly centrifuged, aliquoted and stored at -80°C. The virus stock was titrated by plaque assay on pre-seeded confluent monolayers of MDCK cells. The LD_50_ (half-lethal dose of virus needed to kill mice) was determined in 6-8 weeks old naïve female BALB/c mice using serial dilutions of the virus.

### Recombinant trimeric HA for ELISA

Recombinant HA derived from the A/Michigan/45/2015 H1N1 virus, which is closely related to IVR-180, was produced in mammalian cells. The transmembrane domain of HA gene (AA 18-522) was removed and a CD5 secretion signal was fused to the truncated HA with a SA linker (design shown in **Supplementary Figure 3**). A GCN4-derived trimerizing domain was fused with HA gene by a GSG linker at the C-terminal, along with addition of two tags for affinity purification (HIS and Strep tags spaced by SA linkers). This construct was cloned into a pcDNA3.1 plasmid vector and transfected into pre-seeded HEK293T cells using lipofectamine 2000 reagent. After 24 hr of transfection, the media was replaced with DMEM supplemented with a medium consisting of insulin, transferrin and selenium (ITS media). Culture supernatant was harvested after 48 hr, and appropriate dilution was used for direct coating of Nunc Maxisorp ELISA plates.

### Mouse model

These studies were all performed on 6–8-week-old BALB/c mice strains obtained from Charles River Laboratories, MA. The mice were housed with food and water ad libitum in a pathogen-free animal facility at Icahn School of Medicine at Mount Sinai. Mice were vaccinated intramuscularly (50μl each hind leg, 100μl total per mouse) and infected intranasally (in 50μl PBS per mouse) under ketamine/xylazine anesthesia. All procedures were approved by the Icahn School of Medicine at Mount Sinai Institutional Animal Care and Use Committee (IACUC -2013-1408).

### qRT-PCR

Murine blood was collected for each animal at different time points (as outlined in **Figure 3A**) in 500μl RNA later. The blood was further processed to collect RNA using Ribopure mouse blood RNA Kit (Invitrogen), according to manufacturer’s protocol. 1μg RNA was transcribed into cDNA using cDNA synthesis kit. The cDNA was used for analysis by qRT-PCR using ISG15 primers (forward- GGTGTCCGTGACTAACTCCAT and reverse-TGGAAAGGGTAAGACCGTCCT). GAPDH primers (forward-TGTGTCCGTCGTGGATCTGA and reverse-CCTGCTTCACCACCTTCTTGAT) were used as internal control to calculate the fold induction in ISG15 expression from different groups.

### Serum collection for serology

Murine Blood was collected *via* submandibular bleed 4 weeks post vaccination. The blood was allowed to clot for at 4°C for overnight and serum was collected after centrifugation. The serum was heat inactivated at 56°C for 30 min and stored at -20°C till further use.

### Hemagglutination inhibition assay (HAI)

The sera were treated with receptor destroying enzyme (RDE) for 24 hr at 37°C, followed by incubation with 1.5% sodium citrate at 56°C for 30 min. Thus obtained 1:10-diluted serum samples were further serially diluted 2-fold in V-bottom 96-well plates followed by addition of 4HA units per well of whole IVR-180 virus. The sera-virus mixture was incubated with 0.5% chicken red blood cells (Lampire Biological Laboratories, USA) for 30 min at 4°C or until the blood coagulated. The results were recorded in terms of HAI titers.

### ELISA

Mice sera were further tested for vaccine-specific IgG titers. ELISA plates (Nunc MAXISORP, Thermofisher) were coated with culture supernatant containing recombinant HA, equivalent to 2.5µg H1N1-HA/ml, in bicarbonate buffer and left overnight at 4°C. Plates were washed three times with washing buffer [1X PBS + 0.1% Tween20 (Sigma Aldrich)] and incubated in blocking buffer (5% fat-free milk in PBST) for 1 hr at room temperature. The serum samples were 3-fold serially diluted starting with 1:100 dilution in blocking buffer and allowed to bind HA coated ELISA plates for overnight at 4°C. Next day, the plates were washed three times with washing buffer and incubated with 1:5000 diluted secondary total IgG (Sigma Aldrich) or IgG1 or IgG2a antibodies, conjugated to horse radish peroxidase (HRP) for 1h at room temperature. Finally, the plates were washed three times in washing buffer and incubated with 100µl of tetramethylbenzidine substrate (TMB, BD) until blue color appeared. The colorimetric reaction was stopped by adding 50µl of 1M H_2_SO_4_ and absorbance was measured at 450nm and 650nm wavelengths.

### Antibody-dependent cell cytotoxicity (ADCC)

The ADCC assay was performed following methodology as previously described (35, 36). Briefly, 25000 MDCK/HA (cH1/6) or NA-expressing MDCK cells per well were seeded in a white polystyrene 96 well plate and incubated with heat-inactivated serum, 3-fold serially diluted starting with 1:20, for 4 hr at 37°C. Following the incubation, 75000 FcγRIV-expressing Jurkat cells (Promega), consisting of the Luciferase gene under NFAT promoter, were added in each well and further incubated for 6 hr at 37°C. NFAT binds to NFAT promoter in response to crosslinking of FcγR to immune complexes such as IgG2a, resulting in luciferase gene expression. The luminescence was detected using Bio-Glo luciferase substrate for 15 minutes and the plates were read on Synergy H1 hybrid multimode microplate reader (BioTek). The area under the curve was calculated for each sample from luminescence-versus-dilutions curve.

### ELISPOTS

Spleens were harvested from all animals 10 days post vaccination and collected in RPMI 1640 media supplemented with 10% FBS and 1X penicillin/streptomycin. Single cell splenocyte suspensions were prepared by homogenizing the spleens against a 70 μm strainer. IFN-γ or IL-4 ELISPOT assays were performed using 10^5^ splenocytes/well in a 96-well Polyvinylidene difluoride (PVDF) ELISPOT plates, pre-coated with IFN-γ or IL-4 capture antibody. Splenocytes were unstimulated or restimulated with hemagglutinin (HA-H1N1) overlapping 15-mer peptides (Miltenyi) or whole IVR-180 H1N1 virus and incubated overnight at 37°C, 5% CO2. Cells were then removed from the plates and thoroughly washed in the washing buffer. The wells were incubated with 100 μl of biotinylated polyclonal detection antibody against IFN-γ or IL-4 for 1.5 hr at room temperature (RT). The plates were washed again three times in the washing buffer and incubated with strep-HRP conjugated antibody for 1 hr at RT. Following washing in wash buffer, the plates were incubated with the substrate for 1 hr at RT in dark. Finally, the plates were thoroughly washed under tap water five times and allowed to air dry overnight at RT in dark. The number of spots in each well were manually counted using a dissection microscope and represented as number of IFN-γ or IL-4 producing splenocytes per million splenocytes.

### Virus challenge

1.8x10^4^ plaque forming units (PFU), corresponding to 100LD_50_ of IVR-180, was used for intranasal infection per animal, in a final volume of 50µL per mouse in 1X PBS. The challenge was performed under mild ketamin/xylazine (150μl per mouse; intraperitoneal) sedation as recommended by AICUC. The unvaccinated and unchallenged group was referred to as mock for reference in the study. Body weights were recorded every day to assess the morbidity post-infection until lung harvest. The lungs were collected at 5 days post-infection (DPI) in 1ml 1X PBS, homogenized and the lysate was stored at -80°C until further assays.

### Plaque assay

Plaque assays were performed to titrate the replicating virus in lung of vaccinated versus unvaccinated mice after virus challenge. The lung homogenate was 10-fold serially diluted starting from 1:10 dilution in 1X PBS and incubated on pre-seeded confluent monolayers of MDCK cells for 1 hr at 37°C, 5% CO2 with gentle shaking every 10 min. The diluted samples were then removed, and the monolayers were briefly washed with 1ml 1X PBS. Finally, 1ml of the overlay mixture (2% oxoid agar and 2X minimal essential medium (MEM) supplemented with 1% diethyl-aminoethyl (DEAE)-dextran and 1 μg/ml tosylamide-2-phenylethyl chloromethyl ketone (TPCK)-treated trypsin) was added on top of the monolayers and incubated for 48 hr at 37°C, 5% CO2. The plates were fixed in 4% formaldehyde and immune-stained with IVR-180-postchallenge polyclonal serum. The number of plaques were counted and represented as plaque forming units (PFU)/ml.

### Software

The schematic figures were created with BioRender.com. GraphPad Prism version 9 was used for data visualization and analysis.

## Data availability statement

The original contributions presented in the study are included in the article/**Supplementary Material**. Further inquiries can be directed to the corresponding author.

## Ethics statement

The animal study was reviewed and approved by Institutional Animal Care and Use Committee (IACUC) at the Icahn School of Medicine at Mt Sinai.

## Author contributions

Conceptualization and design, SJ, MS, and BG. Methodology, SJ (viruses, infections, mouse immunization and *in vitro* serological assays), RR (infections, plaque assays), AC, GL (virus preparation and vaccine dose optimization) SJ, and MS (ELISPOTs). Reagents, LC, AC, GL, AG-S, SY, RR, YC, and BG. Investigation and data analysis, SJ, GL, and MS. First draft of the manuscript, SJ and MS. Manuscript review and editing, all authors. Funding acquisition, AG-S, BG, and MS. All authors contributed to the article and approved the submitted version.

## Funding

This study was also partly funded by CRIPT (Center for Research on Influenza Pathogenesis and Transmission), a NIH NIAID funded Center of Excellence for Influenza Research and Response (CEIRR, contract number 75N93021C00014), by the NIAID funded SEM-CIVIC consortium to improve influenza vaccines (contract number 75N93019C00051), by NIAID grant P01AI097092, and by the JBP foundation and the OPP (research grant 2020-215611 (5384)) to AG-S. By NIH/NIAID CEIRS HHSN272201400008C (LC), NIH/NIAID 1R21AI146529 (LC), and NIH/NIAID R21AI151229 (MS). BG acknowledges funding from the European Research Council (ERC) under the European Union’s Horizon 2020 research and innovation program (grant N 817938). SY is supported by The Swiss National Science Foundation (Postdoc Mobility Grant Project ID: P400PB_199292).

## Conflict of interest

The AG-S laboratory has received research support from Pfizer, Senhwa Biosciences, Kenall Manufacturing, Avimex, Johnson & Johnson, Dynavax, 7Hills Pharma, Pharmamar, ImmunityBio, Accurius, Nanocomposix, Hexamer, N-fold LLC, Model Medicines, Atea Pharma and Merck, outside of the reported work. AG-S has consulting agreements for the following companies involving cash and/or stock: Vivaldi Biosciences, Contrafect, 7Hills Pharma, Avimex, Vaxalto, Pagoda, Accurius, Esperovax, Farmak, Applied Biological Laboratories, Pharmamar, Paratus, CureLab Oncology, CureLab Veterinary, Synairgen and Pfizer, outside of the reported work. AG-S is inventor on patents and patent applications on the use of antivirals and vaccines for the treatment and prevention of virus infections and cancer, owned by the Icahn School of Medicine at Mount Sinai, New York. The MS laboratory received research support from ArgenX and Moderna Therapeutics.

The remaining authors declare that the research was conducted in the absence of any commercial or financial relationships that could be construed as a potential conflict of interest.

## Publisher’s note

All claims expressed in this article are solely those of the authors and do not necessarily represent those of their affiliated organizations, or those of the publisher, the editors and the reviewers. Any product that may be evaluated in this article, or claim that may be made by its manufacturer, is not guaranteed or endorsed by the publisher.

## Supplementary material

Supplementary Figure 1 SDI+nanogel and IMDQ-PEG-Chol, individual or combined, define IgG subtype profile: Area under curve was calculated from OD_450_ ELISA values for individual mouse (n=4/group). **(A)** Serum ELISA four weeks post-prime, **(B)** Area under curve eight weeks post-prime and 4 weeks post boost for half of the mice as indicated. ELISA was performed with 3-fold serum dilutions starting with 1:100. Each group presents symbols representing each animal and are represented as geometric mean ± geometric SD.

Supplementary Figure 2
*In vitro* transcribed SDI RNA potently induces IFN-β luciferase activity. HEK293T cells were or transfected with SDI-RNA, before and post treatment with RNase A, along with IFN-β-Luc reporter and pSV-Rluc as an internal control. Live Sendai virus (SeV) was used to infect HEK293T cells and served as a positive control for the assay. The results are represented as fold induction in luciferase activity compared with mock. Statistical analysis was performed using two-sided unpaired T-test and p-values are calculated in reference to the signal for cells transfected with SDI-RNA.

Supplementary Figure 3 Schematic representation of recombinant trimeric hemagglutinin produced for ELISA coating.
