# Supplementary figures and images for: RIG-I and TLR-7/8 agonists as combination adjuvant shapes unique antibody and cellular vaccine responses to seasonal influenza vaccine
Source: Front Immunol. 2022 Nov 8;13:974016. doi: 10.3389/fimmu.2022.974016 (PMC9679288; doi:10.3389/fimmu.2022.974016)

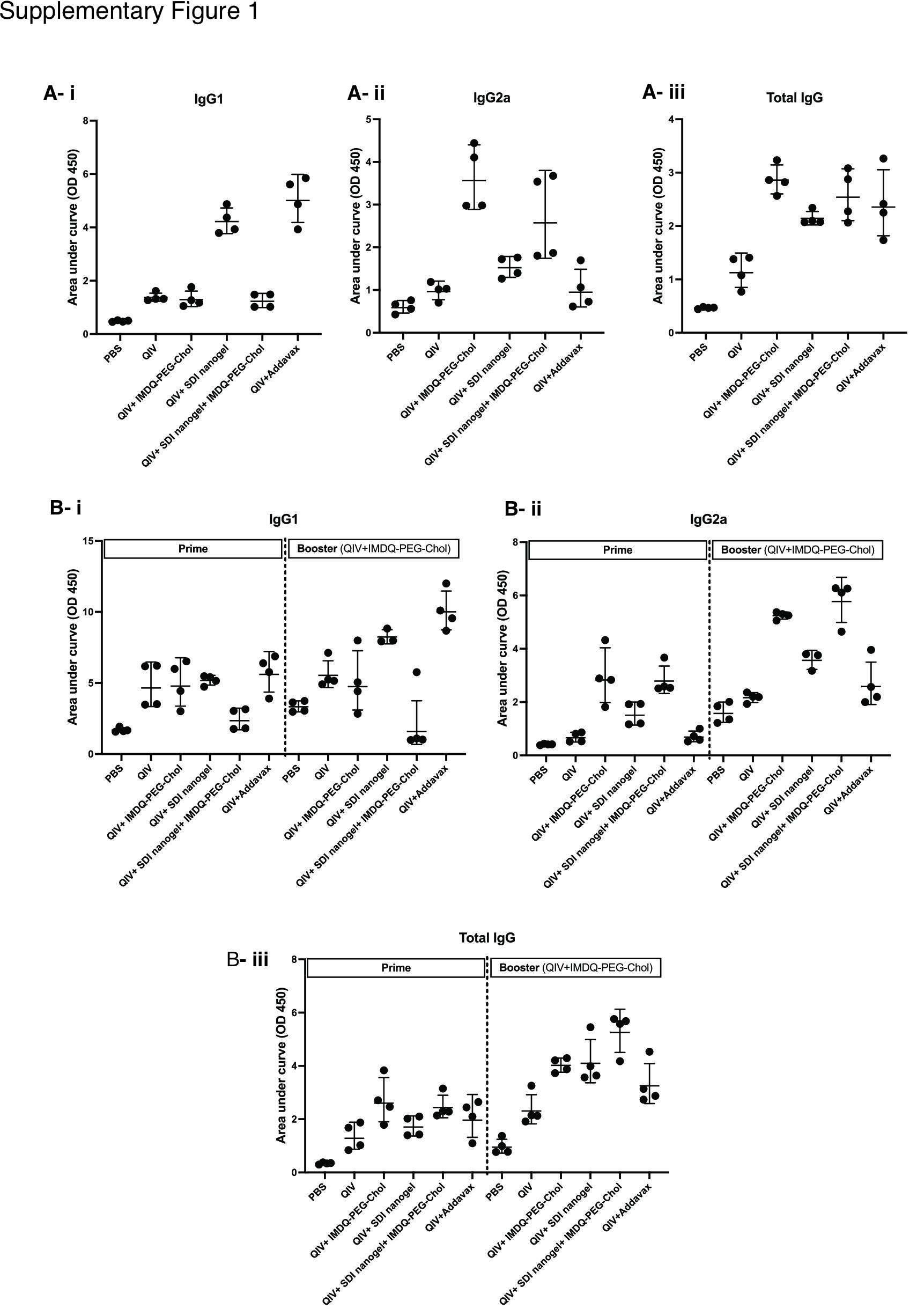

Supplement: Supplementary Figure 1 — SDI+nanogel and IMDQ-PEG-Chol, individual or combined, define IgG subtype profile: Area under curve was calculated from OD450 ELISA values for individual mouse (n=4/group). (A) Serum ELISA four weeks post-prime, (B) Area under curve eight weeks post-prime and 4 weeks post boost for half of the mice as indicated. ELISA was performed with 3-fold serum dilutions starting with 1:100. Each group presents symbols representing each animal and are represented as geometric mean ± geometric SD. [file Image_1.jpeg]

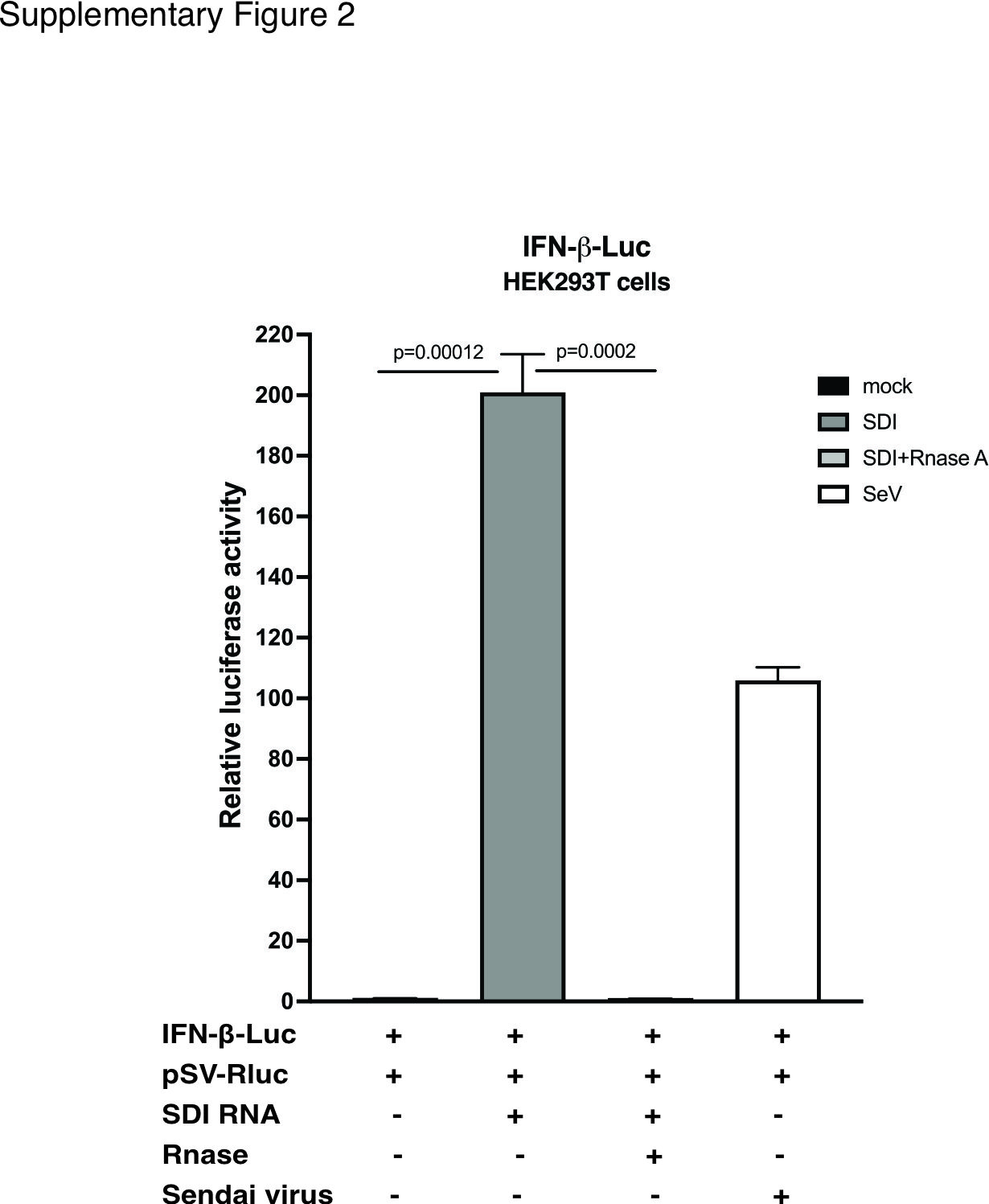

Supplement: Supplementary Figure 2 — In vitro transcribed SDI RNA potently induces IFN-β luciferase activity. HEK293T cells were or transfected with SDI-RNA, before and post treatment with RNase A, along with IFN-β-Luc reporter and pSV-Rluc as an internal control. Live Sendai virus (SeV) was used to infect HEK293T cells and served as a positive control for the assay. The results are represented as fold induction in luciferase activity compared with mock. Statistical analysis was performed using two-sided unpaired T-test and p-values are calculated in reference to the signal for cells transfected with SDI-RNA. [file Image_2.jpeg]

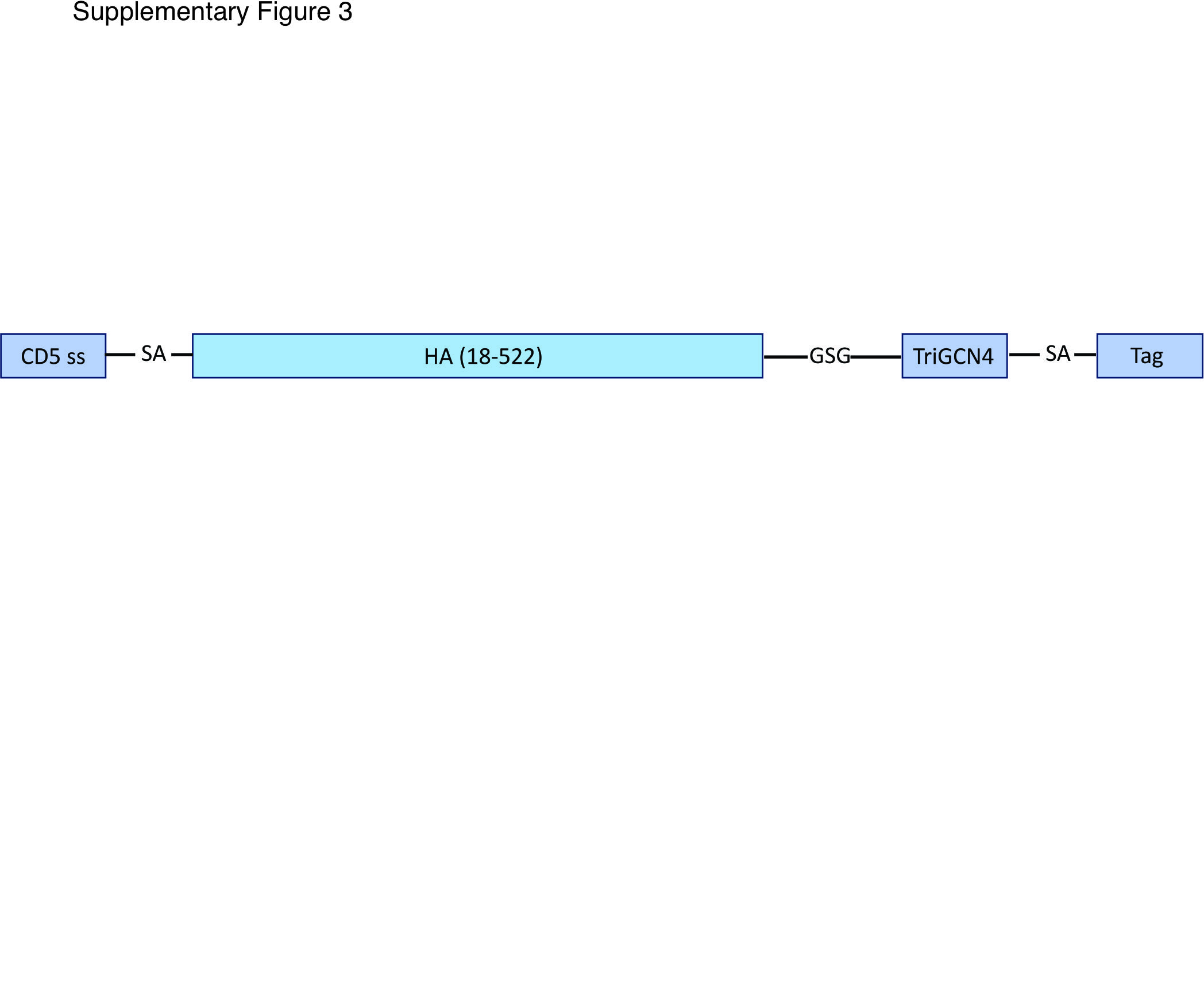

Supplement: Supplementary Figure 3 — Schematic representation of recombinant trimeric hemagglutinin produced for ELISA coating. [file Image_3.jpeg]
